# Synergistic Optimization of Polymer–Surfactant Binary Flooding for EOR: Core-Scale Experimental Analysis of Formulation, Slug Design, and Salinity Effect

**DOI:** 10.3390/polym17162166

**Published:** 2025-08-08

**Authors:** Wenjie Tang, Patiguli Maimaiti, Hongzhi Shao, Tingli Que, Jiahui Liu, Shixun Bai

**Affiliations:** 1 Research Institute of Exploration and Development, Xinjiang Oilfield Company, PetroChina, Karamay 834000, China; sxyttwj@petrochina.com.cn (W.T.); ptgl@petrochina.com.cn (P.M.); shaohongzhi@petrochina.com.cn (H.S.); quetingli1986@petrochina.com.cn (T.Q.); 2 School of Petroleum, China University of Petroleum-Beijing at Karamay, Karamay 834000, China; 2024216866@st.supk.edu.cn

**Keywords:** polymer–surfactant binary flooding, interfacial tension, salinity sensitivity, EOR optimization

## Abstract

As conventional waterflooding enters mid-to-late stages, chemical enhanced oil recovery (EOR) technologies such as polymer–surfactant binary flooding have emerged to address declining recovery rates. This study systematically investigates the synergistic effects of polymer–surfactant binary formulations through core-flooding experiments under varying concentrations, injection volumes, and salinity conditions. The optimal formulation, identified as 0.5% surfactant and 0.15% polymer, achieves a maximum incremental oil recovery of 42.19% with an interfacial tension (IFT) reduction to 0.007 mN/m. A 0.5 pore volume (PV) injection volume balances sweep efficiency and economic viability, while sequential slug design with surfactant concentration gradients demonstrates superior displacement efficacy compared with fixed-concentration injection. Salinity sensitivity analysis reveals that high total dissolved solids (TDS) significantly degrade viscosity, whereas low TDS leads to higher viscosity but only marginally enhances the recovery. These findings provide experimental evidence for optimizing polymer–surfactant flooding strategies in field applications, offering insights into balancing viscosity control, interfacial tension reduction, and operational feasibility.

## 1. Introduction

Polymer–surfactant combination flooding is a common Enhanced Oil Recovery (EOR) method that integrates the complementary mechanisms of polymers and surfactants to achieve superior oil displacement efficiency [1,2,3]. Polymers enhance the viscosity of the injected fluid, improving mobility control and sweep efficiency by mitigating viscous fingering and unfavorable mobility ratios [4,5]. Surfactants, meanwhile, reduce interfacial tension (IFT) between oil and water, enabling emulsification and wettability alteration, which facilitates the detachment of residual oil from rock surfaces [6,7]. The synergy between these components results in a more effective recovery process compared with standalone polymer or surfactant flooding, as it addresses both macroscopic (sweep efficiency) and microscopic (oil mobilization) challenges [8,9]. The performance of polymer–surfactant flooding is influenced by several factors, the general understanding of which is respectively introduced in the following paragraphs.

To begin with, the concentration of polymer in polymer–surfactant flooding plays a critical role in determining viscosity, sweep efficiency, and overall recovery performance. Higher polymer concentrations increase solution viscosity, enhancing mobility control and reducing viscous fingering, thereby improving sweep efficiency [10,11]. However, excessively high concentrations can lead to injectivity challenges due to elevated shear resistance and polymer adsorption on rock surfaces, which may hinder oil displacement [12,13]. The selection of polymer concentration must therefore consider reservoir-specific conditions, such as permeability and salinity, to maximize recovery while minimizing injectivity issues [14,15].

The concentration of surfactants is also a critical factor. Higher surfactant concentrations generally lead to greater reductions in interfacial tension (IFT), which is essential for mobilizing trapped oil [16,17]. However, beyond the critical micelle concentration (CMC), additional surfactant may not further reduce IFT but could increase costs and adsorption losses [18,19]. Surfactant concentration also influences emulsification, a key mechanism for oil displacement. Optimal concentrations promote stable oil-in-water emulsions, improving sweep efficiency [20,21]. The choice of surfactant concentration must balance IFT reduction, emulsification stability, and economic viability [22].

The optimization of injection volume and slug design directly impacts the sweep efficiency and oil recovery. Druetta et al. demonstrate that overlapping polymer and surfactant slugs, with the polymer injected first, enhance viscous oil displacement by improving sweep efficiency [23]. Similarly, Zhao et al. found that sequential injection, starting with polymer followed by surfactant, outperforms combined slugs in heterogeneous reservoirs by improving vertical conformance [24]. Field applications, such as the Mehsana Asset pilot, highlight the importance of tailored slug designs, including a 6-month surfactant slug followed by a 2.5-year polymer chase, which significantly improved oil rates [25]. Simulation studies by Oliveira et al. further support the use of larger slug sizes (0.6 PV) and higher injection rates for optimal recovery [26]. Comparative analyses of sequential versus simultaneous injection strategies reveal trade-offs in performance. While simultaneous injection may reduce operational complexity, sequential injection with optimized slug sizes often achieves higher recovery, particularly in heterogeneous or low-permeability formations [27]. These findings collectively underscore the need for customized injection strategies based on reservoir-specific conditions.

In terms of salinity, high salinity reduces polymer viscosity through charge screening effects and increases surfactant adsorption onto rock surfaces, compromising flood efficiency [28,29]. Khamees et al. demonstrated that high salinity, particularly in the presence of divalent cations (Ca^2+^, Mg^2+^), reduces recovery factors by 15–20% due to polymer viscosity loss and surfactant retention [28]. Similarly, Ghosh et al. reported challenges in carbonate reservoirs, where high salinity exacerbates surfactant precipitation and degrades polymer thermal stability [29]. However, it has been reported that high salinity enhances microemulsion formation, which may in turn increase the oil recovery [30,31]. The effect of salinity should therefore be evaluated on a case-by-case basis.

Reservoir heterogeneity significantly influences the efficiency of polymer–surfactant combination flooding. In high-permeability cores, polymer–surfactant flooding shows pronounced effectiveness due to improved mobility control and interfacial tension reduction. Conversely, low-permeability cores exhibit limited recovery due to restricted pore accessibility and higher surfactant adsorption. Lv et al. further highlight that inter-layer heterogeneity disproportionately affects low-permeability zones, with extreme permeability contrasts reducing overall recovery enhancement [32]. Fingering phenomena, exacerbated by intra-layer heterogeneity, disrupt flood front stability, particularly during waterflooding stages. Zhang et al. note that remaining oil distribution is more affected by fingering in polymer-only slugs compared with polymer–surfactant combinations, as the latter improves sweep efficiency through synergistic effects [33]. Mitigation strategies for heterogeneity challenges include optimizing polymer molecular weight to match reservoir permeability and designing tailored slug sequences to address permeability contrasts. Additionally, combining polymer–surfactant flooding with conformance control techniques, such as gel treatments or selective injection, can further enhance sweep efficiency in highly heterogeneous formations.

The rest of the paper is structured as follows. First, the materials and experimental setup are described. The results are presented, and the effects of potential influencing factors such as polymer and surfactant concentration, the injection volume, slug design, and salinity are analyzed, and an optimized surfactant-polymer formulation is proposed. Subsequently, the key findings and understandings are summarized in the conclusion.

## 2. Materials and Methods

### 2.1. Materials

The experimental oil sample was taken from the outlet of the oilfield production separator, with a viscosity of 61 mPa·s for the dehydrated crude oil at ambient temperature, measured with an OFITE 900 viscometer from OFI Testing Equipment, Inc. (Houston, TX, USA) at 6 rpm. This experiment employed a blended system of crude oil and kerosene, with a density of 0.82 g/cm^3^ and a viscosity of 33.2 mPa·s at normal temperature. The oilfield formation water used was formulated with NaCl (13.278 g/L), CaCl_2_ (0.123 g/L), NaHCO_3_ (3.869 g/L), and MgCl_2_·6H_2_O (0.644 g/L).

The surfactant used was coconut oil diethanolamide and is commercially named 6501, and the polymer was a polyacrylide-type product named 2SP with a molecular weight ranging from 9 to 12 million and a purity of 92.13%. The cores used in the experiment were 30 cm in length and 2.5 cm in diameter, with an average permeability of 200 × 10^−3^ μm^2^ and an average porosity of 20%. Interfacial tensions between the oil and various solutions were measured using a KRUSS SDT spinning drop tensiometer (KRÜSS Scientific Instruments, Hamburg, Germany) at 6000 rpm. Viscosities and interfacial tensions were taken as the average of three measurements.

### 2.2. Core Flooding Procedures

The dimensions, dry weight, and permeability (by N_2_) of the core were first measured. The core was then assembled into a core holder and vacuumed for 4 h. Formation brine was then used to fully saturate the core. Subsequently, the core’s permeability was determined via brine injection, and its wet weight was recorded after complete water saturation. The pore volume and porosity of the core were then calculated. The core was again loaded into the core holder, and crude oil was injected into the core at a low rate of 0.02 mL/min until no more water was produced at the outlet to establish the initial water saturation.

Using a core flooding setup manufactured by Yangzhou Huabao (Yangzhou, China), the flooding was then commenced by water injection at 0.3 mL/min until the water cut at the outlet became 98%, followed by a 0.5 PV (pore volume, subject to changes during optimization of injection volumes) of combination formulation flooding, also at 0.3 mL/min. Water flooding was then resumed until the water cut reached 98% again. The net confining pressure was maintained at 3 MPa throughout the saturation and flooding processes. The volume of the produced oil was recorded based on the true oil phase, and the oil contained in the produced emulsion, if any, was neglected on the assumption that emulsified oil is negligible compared with bulk oil and that such neglect does not fundamentally change the trend of recoveries with changes in varying factors.

## 3. Results and Discussion

### 3.1. Impact of Surfactant Concentration on EOR Ability of Combination Flooding

**Figure 1:**
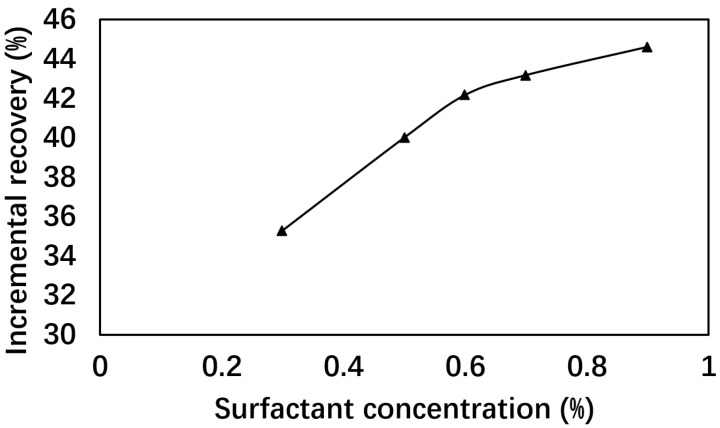
Incremental recoveries at different surfactant concentrations with polymer concentration fixed at 0.15%.

To analyze the impact of surfactant concentration on EOR performance, five sets of long core flooding tests were conducted at fixed polymer concentration (0.2%) and varying surfactant concentrations. The results are listed in Table 1 and Figure 1.

The incremental oil recovery (additional recovery due to flooding agent injection and subsequent water flooding) increased from 35.28% to 45.63% as surfactant concentration rose from 0.3% to 0.9%, with a critical turning point at 0.5%. Notably, the viscosity increased from 108 mPa·s to 147 mPa·s with increasing surfactant concentration, indicating the co-participation of surfactant and polymer molecules in the inter-molecular network formation [34].

While higher surfactant concentrations yield better performance, the marginal gain above 0.5% surfactant does not justify the associated costs. This highlights the need for cost-effective formulations, as advocated by Joshi et al. [35]. Considering the economics and feasibility of oilfield use, a surfactant concentration of 0.5% was selected for subsequent experiments.

### 3.2. Influence of Polymer Concentration on EOR Efficiency of Combination Flooding

**Figure 2:**
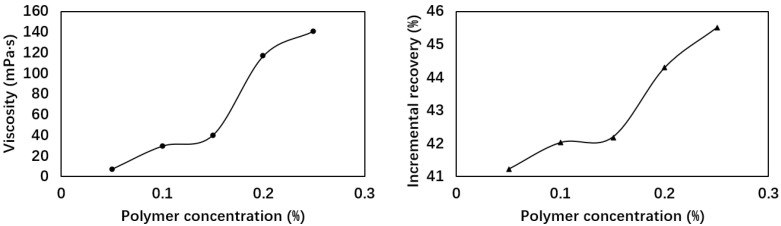
Viscosities of 2SP polymer at different concentrations and the corresponding incremental recoveries.

Five sets of long core flooding tests were conducted to investigate the impact of polymer concentration on the EOR ability of the combination flooding at a fixed surfactant concentration (0.5%). The results are listed in Table 2 and Figure 2.

Figure 2 illustrates the relationship between polymer concentration and viscosity (left panel) as well as the corresponding incremental oil recoveries (right panel). As shown in Table 2, when the surfactant concentration was fixed at 0.5%, the viscosity of the binary system increased from 7.3 mPa·s to 141 mPa·s with increasing polymer concentration from 0.05% to 0.25%. The incremental oil recovery (binary flooding + post-waterflooding) exhibited a gradual increase from 41.22% to 45.51%. The observed trend can be attributed to the dual roles of polymer and surfactant in the binary system. At low polymer concentrations (<0.15%), the dominant factor is the reduction in IFT, which enhances displacement efficiency by lowering capillary forces and improving wettability alteration. By comparison, at high polymer concentrations (>0.15%), viscosity becomes the primary driver, increasing sweep efficiency through reduced mobility ratios. However, considering the cost-effectiveness and the potential injectivity issues at higher polymer concentrations for field application, the concentration of 0.15% was selected for subsequent tests.

### 3.3. Influence of Injection Volume on Incremental Recovery

**Figure 3:**
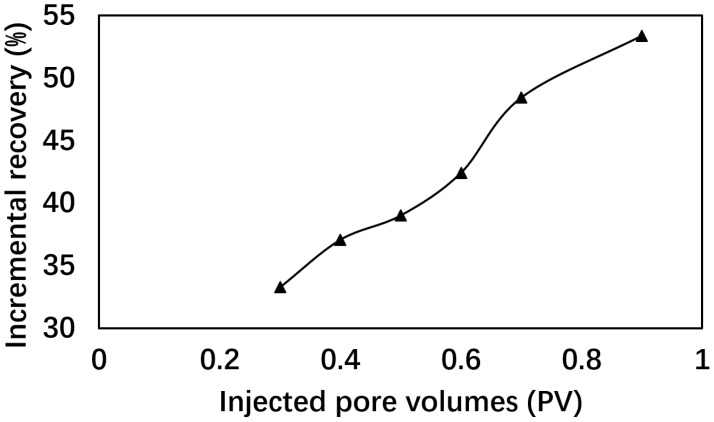
Incremental recoveries at different injected volumes.

To investigate the impact of injection volume on incremental oil recovery, a series of core flooding experiments was conducted under a fixed formulation of surfactant (6501) concentration at 0.5% and polymer (2SP) concentration at 0.15%. The total injection volumes ranged from 0.3 to 0.9 PV. The results are summarized in Table 3 and Figure 3, which illustrates the incremental recovery efficiency as a function of PV.

**Figure 4:**
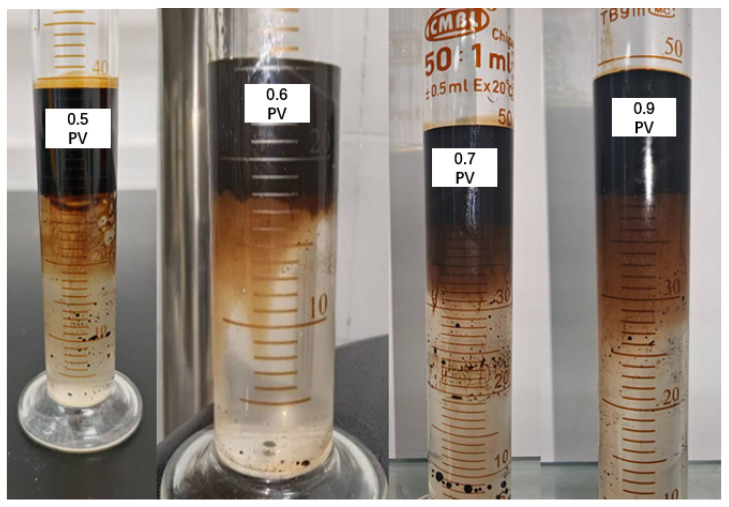
Emulsification of effluent at different injection volumes.

The data reveal an almost linear relationship between injection volume and incremental recovery. Notably, the viscosity remains constant at 40.2 mPa·s across all injection volumes, implying that polymer concentration is not the limiting factor for mobility control. Another phenomenon with increasing injection volume is the progressive emulsification in the effluent shown in Figure 4. Emulsion volume peaks at 0.9 PV, correlating with the highest incremental recovery. This suggests that emulsification also contributed to the incremental recovery. Below 0.5 PV, emulsion was not observed, which is likely due to the dilution by the formation brine, which lowered the effective surfactant and polymer concentrations. The dilution effect became less significant with increasing ratio of the formulation to formation brine. However, economic considerations and pressure constraints necessitate optimizing the injection volume. While higher volumes improve recovery, they also escalate operational costs and risk formation damage due to elevated pressure gradients. Thus, an injection volume of 0.5 PV emerges as the optimal balance between performance and practicality.

### 3.4. Comparison Between Different Slug Designs

The efficacies of slug design strategies were investigated and compared, which include concentration-gradient slugs (both forward and reverse gradients) for surfactant and polymer, respectively. The baseline formulation is 0.5% 6501 and 0.15% 2SP as determined from previous sections, and for gradient slugs, three schemes were tested as follows:

Forward Gradient: Increasing surfactant concentration (0.3%, 0.5%, 0.7%, 0.4%, 0.5%, and 0.6%) at constant polymer concentration (0.15%).

Reverse Gradient: Decreasing surfactant concentration (0.7%, 0.5%, 0.3%, 0.6%, 0.5%, and 0.4%) at constant polymer concentration (0.15%).

Polymer Gradient: Increasing polymer concentrations (0.12%, 0.15%, 0.18%) at a fixed surfactant concentration (0.5%).

**Figure 5:**
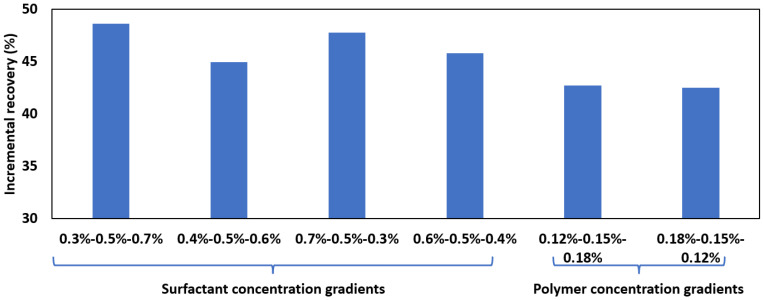
Recoveries at different slug designs.

The incremental recoveries achieved by each slug design are summarized and compared in Table 4 and Table 5 and Figure 5. It should be noted that all gradient designs share the same average chemical usage as the baseline formulation; therefore, any differences in the recoveries should be due to the injection schemes. The baseline system (0.5% 6501 + 0.15% 2SP) achieved an incremental recovery of 42.19%, while both the forward and reverse surfactant gradients led to higher recoveries (Table 4). A few possible mechanisms may have led to this result. First, a higher surfactant concentration is conducive to the mobilization of more oil, some of which may even be emulsified, which further contributes to the oil mobilization. Not only does this explain the higher recoveries achieved by gradient–surfactant slugs, but it also justifies the higher recovery obtained by the 0.7, 0.5, 0.3 combination (47.78% and 48.64%) compared with the 0.6, 0.5, 0.4 combination (45.81% and 44.93%). The order of gradient, namely, the forward and reverse injection schemes, did not exhibit any significant differences, possibly due to the relatively small amount of injection for each slug, which becomes readily mixed within the cores during injection.

Surfactant-gradient schemes consistently outperform polymer-gradient ones, with incremental recoveries exceeding 45% compared with less than 42% for polymer-gradient systems. This confirms that surfactant concentration plays a dominant role in reducing interfacial tension and enhancing oil mobilization, whereas polymer adjustments primarily affect flow profile optimization, and that an inconsistent polymer concentration jeopardizes the profile-controlling effect.

### 3.5. Impact of Salinity on the EOR Performance

To investigate the effect of salinity on the performance of the polymer–surfactant binary system, four sets of flooding tests were conducted with the formulation prepared with different salinities (Table 6). All experiments were conducted using the optimal formulation (0.5% 6501 + 0.15% 2SP) with a total injection volume of 0.5 PV.

It can be seen from Table 6 that, as salinity increased, the viscosity of the formulation decreased significantly (e.g., from 53.4 mPa·s at half the original salinity to 27.6 mPa·s at S = 5.3 times the original salinity), leading to reduced mobility control and lower incremental oil recovery. It has been mentioned in the literature that higher salinity can contribute to the formation of more middle-phase microemulsion, which solubilizes more oil; however, in this work, no apparent microemulsion was observed in the effluent, possibly due to the dilution effect by the formation water, as mentioned earlier. In any case, high salinity did not lead to higher recoveries but rather severely compromised the effectiveness of the formulation by reducing viscosity and surface activity. Low salinity leads to a similar recovery (44.98%) to the baseline formulation (45.30%), indicating that the viscosity at the original salinity, 40.2 mPa·s, is sufficient to compensate for the low level of heterogeneity of the core, and higher viscosity values do not bring additional benefits. These findings underscore the importance of conducting salinity scans during field applications to optimize chemical flooding design.

## 4. Conclusions

In this study, a polymer–surfactant binary flooding formulation was optimized through comprehensive core flooding experiments, and the key insights are as follows:

- The optimal formulation (0.5% surfactant + 0.15% polymer) achieves synergistic benefits: elevated viscosity (40.2 mPa·s) enhances mobility control, while reduced IFT (0.007 mN/m) improves microscopic displacement.
- The 0.5 PV injection volume represents a practical compromise between incremental recovery and operational constraints such as injectivity.
- Sequential slug design with surfactant gradients outperforms fixed-concentration injection, highlighting the importance of tailored slug sequences.
- High salinity reduces viscosity and does not lead to emulsification at the effluent, whereas low salinity increases the viscosity without contributing to higher recovery, possibly due to the insignificant level of heterogeneity of the cores.

These results underscore the necessity of salinity screening in field implementations to mitigate adverse effects on formulation performance. Future research should explore advanced slug designs, such as dynamic concentration adjustments, and incorporate reservoir heterogeneity to further optimize polymer–surfactant flooding strategies. 

## Figures and Tables

**Table 1:** Recoveries with different surfactant concentrations.

Formulation	Viscosity (mPa·s)	IFT (10^−3^ mN/m)	Incremental Recovery (%)
0.3% S + 0.2% P	107.68 ± 0.45	9.13 ± 0.27	35.28
0.5% S + 0.2% P	117.17 ± 0.23	7.11 ± 0.02	42.19
0.6% S + 0.2% P	123.86 ± 0.14	6.57 ± 0.11	43.48
0.7% S + 0.2% P	132.10 ± 0.21	5.14 ± 0.04	44.32
0.9% S + 0.2% P	147.22 ± 0.20	1.89 ± 0.04	45.63

**Table 2:** Recoveries with different polymer concentrations.

Formulation	Viscosity (mPa·s)	IFT (10^−3^ mN/m)	Incremental Recovery (%)	Peak Pressure (MPa)
0.5% S + 0.05% P	7.34 ± 0.08	7.17 ± 0.03	41.22	0.75
0.5% S + 0.1% P	27.92 ± 0.04	7.27 ± 0.04	42.03	0.88
0.5% S + 0.15% P	40.18 ± 0.03	7.12 ± 0.14	42.19	1.35
0.5% S + 0.2% P	117.17 ± 0.23	7.11 ± 0.02	44.30	1.91
0.5% S + 0.25% P	141.42 ± 0.41	8.13 ± 0.33	45.51	1.93

**Table 3:** Recoveries with different injection volumes.

PV	Viscosity (mPa·s)	IFT (10^−3^ mN/m)	Incremental Recovery (%)	Peak Pressure (MPa)
0.3	40.18 ± 0.03	7.12 ± 0.14	34.27	1.21
0.4	40.18 ± 0.03	7.12 ± 0.14	39.07	1.27
0.5	40.18 ± 0.03	7.12 ± 0.14	42.19	1.35
0.6	40.18 ± 0.03	7.12 ± 0.14	45.42	2.16
0.7	40.18 ± 0.03	7.12 ± 0.14	48.43	2.34
0.9	40.18 ± 0.03	7.12 ± 0.14	53.38	3.11

**Table 4:** Recoveries using different slug designs with forward and reverse surfactant gradients.

Formulation	Incremental Recovery (%)
0.7% S + 0.15% P (0.15 PV)	
0.5% S + 0.15% P (0.15 PV)	47.78
0.3% S + 0.15% P (0.15 PV)	
0.3% S + 0.15% P (0.15 PV)	
0.5% S + 0.15% P (0.2 PV)	48.64
0.7% S + 0.15% P (0.15 PV)	
0.6% S + 0.15% P (0.15 PV)	
0.5% S + 0.15% P (0.2 PV)	45.81
0.4% S + 0.15% P (0.15 PV)	
0.4% S + 0.15% P (0.15 PV)	
0.5% S + 0.15% P (0.2 PV)	44.93
0.6% S + 0.15% P (0.15 PV)	

**Table 5:** Recoveries using different slug designs with forward and reverse polymer gradients.

Formulation	Incremental Recovery (%)
0.5% S + 0.18% P (0.15 PV)	
0.5% S + 0.15% P (0.2 PV)	42.48
0.5% S + 0.12% P (0.15 PV)	
0.5% S + 0.12% P (0.15 PV)	
0.5% S + 0.15% P (0.2 PV)	42.74
0.5% S + 0.18% P (0.15 PV)	

**Table 6:** Recoveries with the binary formulation prepared at different salinities.

Formulation	Viscosity (mPa·s)	Incremental Recovery (%)
Original salinity	40.18 ± 0.03	45.30
2.7 × salinity	30.45 ± 0.27	40.46
5.3 × salinity	27.61 ± 0.14	41.52
0.5 × salinity	53.22 ± 0.44	44.98

## Data Availability

Data inquiries can be directed to the corresponding author.

## Abbreviations

The following abbreviations are used in this manuscript:

IFT	Interfacial tension
PV	Pore volume
EOR	Enhanced oil recovery
TDS	Total dissolved solids
